# Microstructural Evolution and Mechanical Performance of A500 Bulletproof Steel Joints Welded with Austenitic and Ferritic Filler Materials

**DOI:** 10.3390/ma18050929

**Published:** 2025-02-20

**Authors:** Mert Bircan, Kaiyang Pan, Hongshan Zhao, Jianwen Fan, Han Dong

**Affiliations:** 1 State Key Laboratory of Advanced Special Steel, School of Materials Science and Engineering, Shanghai University, Shanghai 200444, China; mertcan.bircan@cvsair.com.tr (M.B.); pankaiyanger@163.com (K.P.); fanjw_nwpu@aliyun.com (J.F.); 13910077790@163.com (H.D.); 2 Zhejiang Institute of Advanced Materials, Shanghai University, Jiaxing 314100, China

**Keywords:** bulletproof steel, mechanical properties, microstructure, welding joints, ballistic resistance

## Abstract

This study examines the microstructural evolution and mechanical properties of A500 bulletproof steel joints welded with austenitic stainless steel (ER371) and ferritic (T91) filler materials. While austenitic fillers are traditionally used in bulletproof steel welding to prevent cracking and hydrogen embrittlement, their lower hardness creates a potential weakness in welded joints. This research explores an alternative approach using a newly developed ferritic filler material to achieve strength matching with the base material. Detailed microstructural characterization was conducted using Optical Microscopy (OM) and Scanning Electron Microscopy (SEM), while mechanical properties were evaluated through tensile testing, impact testing, and hardness measurements. The results revealed significantly different mechanical behaviors between the two filler materials, with the ferritic filler achieving superior weld metal hardness (470 HV1) compared to the austenitic filler (185 HV1) in WZ. The fine-grained heat-affected zone (FGHAZ) exhibited the highest hardness (518 HV1) in A500-T91 joints and (480 HV1) in A500-ER371 joints, while ballistic testing demonstrated enhanced penetration resistance with the ferritic filler material.

## 1. Introduction

Bulletproof steels have a wide range of applications in civilian and military fields, providing protection for vehicles and buildings due to their high ballistic resistance [1]. Welding plays a crucial role in determining the processing and ballistic performance of bulletproof steel. Currently, austenitic stainless steel filler materials are commonly used, as they enhance toughness and prevent hydrogen embrittlement [2,3]. Additionally, these commercial austenitic fillers are designed with high austenite stability to prevent deformation-induced martensitic transformation, which could otherwise lead to reduced toughness and increased susceptibility to hydrogen embrittlement [4]. However, these filler materials result in welds with weaker strength matching, failing to provide the same level of ballistic protection as the base metal (BM). Consequently, developing a filler material that can effectively enhance weld hardness and improve ballistic performance is of great importance [5,6].

The main challenge in welding armor steels is their high carbon equivalent (Ceq). Gas Metal Arc Welding (GMAW) is predominantly used for armored vehicles due to its high deposition rate and industrial applicability [7,8,9]. However, the welding heat cycle causes softening in the heat-affected zone (HAZ), resulting in lower strength compared to the base material. The properties of the HAZ are influenced by factors such as filler material, welding method, and heat input. While austenitic filler metals are commonly used in armor steel welds due to their low hydrogen diffusion in the HAZ, their inherent austenitic structure (FCC) results in lower hardness without phase transformation strengthening. In contrast, ferritic filler materials could potentially achieve better strength matching through martensitic transformation during rapid cooling [10,11,12]. Research on achieving strength matching through ferritic filler materials has shown promising potential, but requires further investigation. Saxena et al. [13] demonstrated that welded joints of Armox 500 (Ramor 500) steel using austenitic and ferritic filler achieved only 41% and 31% of the base material’s tensile strength, respectively. Morsy et al. [2] reported HAZ softening in armor steel welded with austenitic wire due to retained austenite formation. However, these studies lack comprehensive analysis of the microstructural evolution and mechanical properties of these materials.

This study investigated the microstructural evolution and mechanical properties of armor steel joints welded with austenitic (ER371) and ferritic (T91) filler metals using the GMAW method. Different regions within the welded joint were subjected to comprehensive microstructural analysis, microhardness measurements, and mechanical testing. Furthermore, ballistic testing was conducted to validate the effectiveness of strength matching achieved through the ferritic filler material.

## 2. Materials and Methods

The material used in this study was 4.0 mm thick bulletproof steel with a hardness rating of 500 HB (named for Guangxi/China/A500), produced through industrial processes. The composition of both the base metal and the welding consumables is detailed in Table 1. The carbon equivalent of the steel was calculated to evaluate its susceptibility to cold cracking, which is a critical factor in armor applications [9]. This low carbon equivalent is advantageous for welding process performance and serves as a critical parameter for optimizing welding conditions, including preheating and post weld heat treatment.

(1) $$Ceq=\%C+\frac{\%Mn}{6}+\frac{\%Cu+\%Ni}{15}+\frac{\%Cr+\%Mo+\%V}{5}$$

According to the formula of the International Institute of Welding (IIW), the carbon equivalent (Ceq) value of A500 steel was found to be approximately Ceq (A500) ≈ 0.679. This showcases the good weldability of the base material, which made it possible to conduct welding experiments with the ferritic filler material [14].

**Figure 1:**
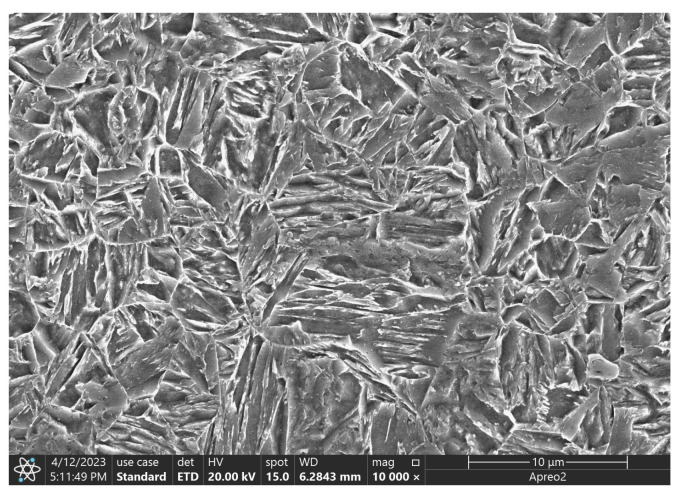
A500 steel (Base Metal/BM) microstructure.

The A500 steel underwent hardening and tempering, resulting in a martensitic microstructure. The SEM image depicting the steel microstructure is presented in Figure 1.

**Figure 2:**
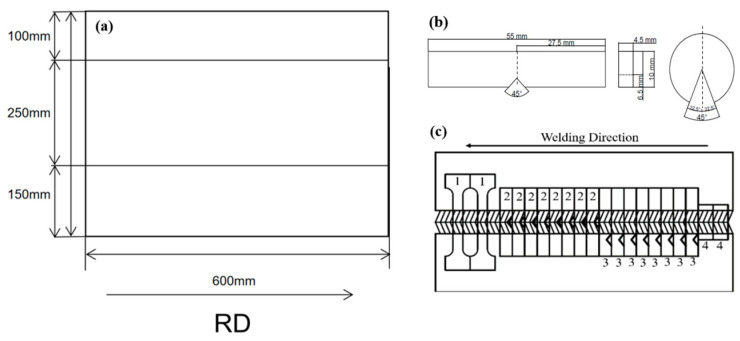
(**a**) Schematic diagram of the cut A500 bulletproof plate. (**b**) Prepared test specimens: 1. Tensile test sample, 2. Impact test welding zone, 3. Impact test HAZ (Heat Affected Zone), 4. Hardness test sample. (**c**) Schematic diagram of the weld mouth.

The A500 steel was cut into three pieces, measuring 100 mm, 250 mm, and 150 mm, respectively, using a (Guangdong/China/DAPENG 6020) laser cutting machine, as shown in Figure 2a. To reduce welding stress and prevent cold cracking, the plates were preheated to 200 °C for one hour in an electric drying oven. Double-sided welding was performed using a (Osaka/Japan/Panasonic Y-500FR2) welding machine, beginning from the groove surface. After completing the initial weld, it was cleaned and ground to remove any pores, and welding continued on the opposite side of the groove. One plate was welded with 1.2 mm ER90S-B9 (T91) ferritic wire, while the other was welded with 1.0 mm ER371 austenitic wire. A ceramic liner was placed behind the weld to prevent penetration and pore formation. Following the welding process, the plates were kept in the electric drying oven at 180 °C for one hour, then cooled to room temperature. The specimens were prepared for the Charpy notch test as shown in Figure 2b. Notch impact details were prepared with 45° weld edges and a V-notch depth of 2 mm. For the Charpy notch test, the materials were prepared with a thickness of 4.0 mm, a length of 55 mm, and a height of 6.5 mm. For the tensile test, two specimens were taken each from A500 steel welded with ER371 and T91 filler materials, respectively, resulting in a total of four specimens tested. X-ray flaw detection was performed to evaluate weld defects, with a grading level of I indicating a successful weld. The welding heat input for both processes was calculated using the following formula:


(2) $$E=\frac{U\times I\times 60\times \eta }{V\times 1000}$$

Here, I represents the welding current amperes (A), U denotes the arc voltage (V), V is the welding speed(mm/min), η indicates the thermal efficiency coefficient, and E represents the heat input (kj/mm). The efficiency of the GMAW method ranges from 0.6 to 0.8. Table 2 list the welding parameters for the two wire types. Frontal welding uses an Ar/CO_2_ shielding gas mixture at a flow rate of 20 L/min and a thermal efficiency of 0.6. The higher heat input from the T91 wire increased welding stresses compared to the ER371 wire, resulting in a slightly smaller angle at the pinch.

Charpy notch impact tests were conducted (aligned in the rolling direction) using an (Norwood, MA, USA/Instron 750MPX) pendulum impact tester. For tensile tests, specimens with a gauge length of 25 mm were tested using an (Norwood, MA, USA/Instron 5982) Tensile Tester at a speed of 2 mm/min. Figure 2c shows the prepared test specimens of the welded base material. Ballistic tests were conducted on A500 bulletproof plates welded with T91 and ER371 welding consumables. SS109 bullets are a variant of the 5.56 mm × 45 mm ammunition standardized by NATO, and are also known as M855 in U.S. military terminology. This ammunition is specifically designed for infantry rifles and light machine guns. SS109 were fired at the plates from a distance of 30 m at a normal incidence angle of 0°. Each welded plate contained two weld seams, with two effective shots fired at each weld seam and within 10 mm of the WZ.

**Figure 3:**
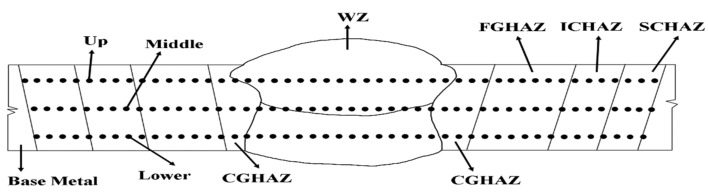
Schematic of hardness test locations at the upper, middle, and lower sections of the weld zone (WZ) and heat-affected zones (HAZ) in the weld cross-section.

Hardness measurements were conducted using a (Pennsylvania/USA/Wilson Hardness VH1102) tester. Table 3 present the hardness measurements of A500 steel, with an average hardness value of 509 HV1 for the bulletproof base material. For the welded materials, hardness tests were performed at the upper, middle, and lower sections, as shown in Figure 3.

As per Volkan’s research, in Table 4, it is observed that the hardness values of Armox 500T steel are similar to those of A500 steel [15].

The microstructures of the welded zones were analyzed using an optical microscope (OM, Oberkochen/Germany/Axio Imager M2m) and a (Billerica, MA, USA/Bruker Apreo 2S) scanning electron microscope (SEM).

**Figure 4:**
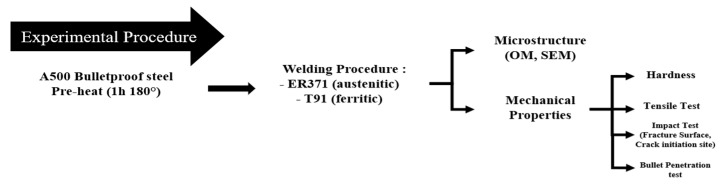
The experimental flow chart summary.

Figure 4 shows the experimental flow chart for the microstructural and mechanical characterization of A500 bulletproof steel welded with austenitic (ER371) and ferritic (T91) filler materials.

## 3. Results and Discussion

### 3.1. Microstructure

**Figure 5:**
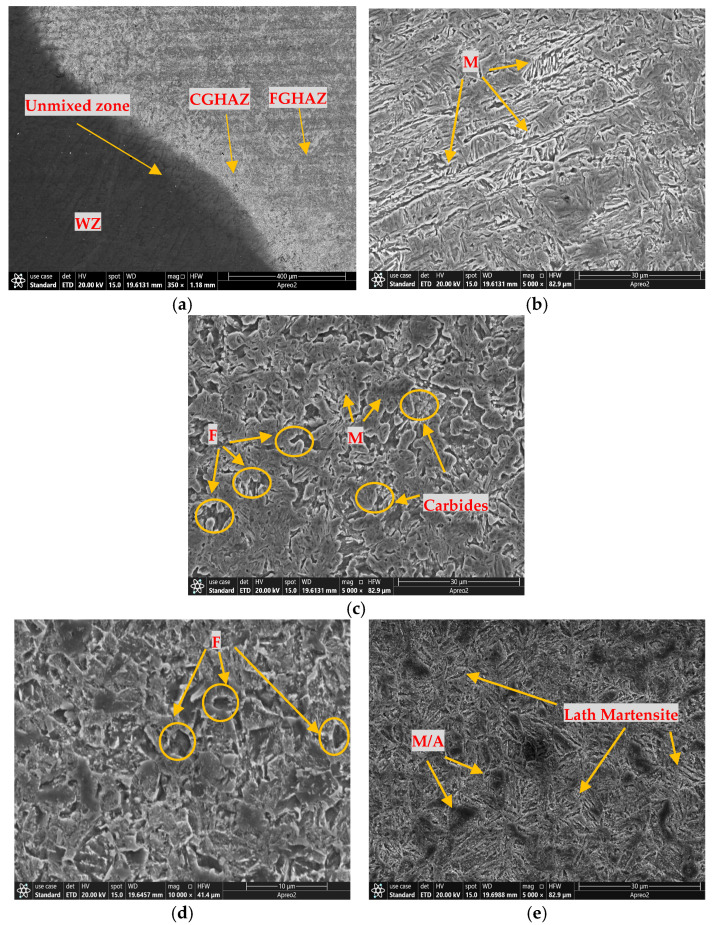
A500-T91 steel SEM of the (**a**) WZ, CGHAZ, and FGHAZ; (**b**) CGHAZ; (**c**) FGHAZ; (**d**) ICHAZ; and (**e**) SCHAZ-BM (M: Martensite, M/A: Martensite/Austenite, F: Ferrite).

To examine the welding performance, microstructure observation of the WZ, Fusion Zone (FZ), and HAZ is essential. The SEM microstructures of the A500-T91 sample are shown in Figure 5. In Figure 5a, it can be observed that the increased heat input during the welding of T91 to the base metal resulted in the formation of a dendritic structure at the boundary line separating the WZ and the Coarse-Grained Heat-Affected Zone (CGHAZ). The CGHAZ is defined as the zone within the HAZ where the grain structure undergoes significant growth due to the high temperatures experienced during welding. Furthermore, in the CGHAZ, a gradual transition of microstructures was observed as it progressed from the coarse-grained zone near the weld interface to the fine-grained zone in the Fine-Grained Heat-Affected Zone (FGHAZ). When examining Figure 5b, it can be seen that due to its proximity to the weld, CGHAZ is exposed to high heat. Lath martensite formation can be observed in this zone. In Figure 5c, light-toned zones are generally associated with ferritic structures. These zones are characterized by low carbon content and often consist of fine-grained ferrite commonly observed in the FGHAZ. Darker zones may represent martensite phases. Martensite phases occur in the FGHAZ at intermediate cooling rates and appear as linear or lamellar structures that are distinctly visible as dark areas in SEM images. In Figure 5e, M/A formation is observed in the Subcritical Heat-Affected Zone (SCHAZ)-BM zone due to cooling of the material. Needle-like structures are typical for martensite, and these structures can be observed in Figure 5e.

**Figure 6:**
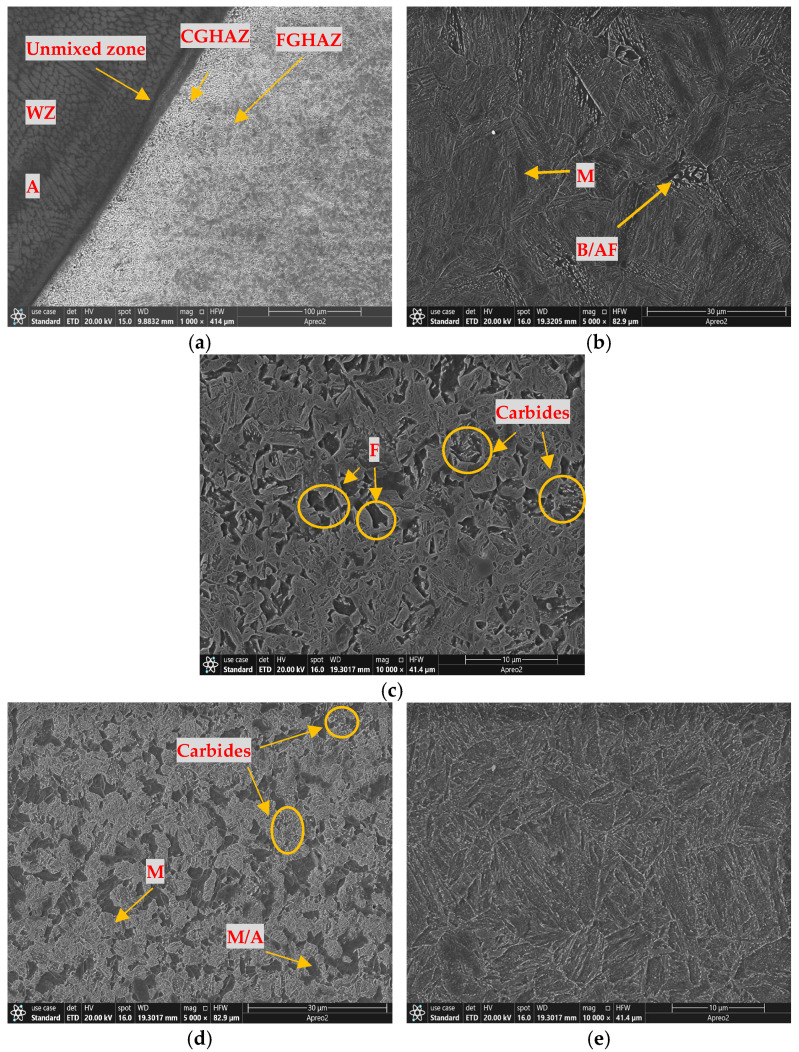
A500-ER371 steel SEM of the (**a**) WZ, CGHAZ, and FGHAZ; (**b**) CGHAZ; (**c**) FGHAZ; (**d**) ICHAZ; and (**e**) SCHAZ-BM (A: Austenite, B/AF: Bainite/Acicular Ferrite).

Volkan observed distinct microstructural features of MIL-A with different filler materials. For welds with ferritic filler material, the microstructure consists primarily of martensite, with some dendritic and bainitic formations. In contrast, welds with austenitic filler material show a characteristic austenitic matrix containing dispersed ferritic structures [15]. The microstructures of the A500-ER371 sample are shown in Figure 6. Figure 6a shows the WZ, CGHAZ, and FGHAZ of the sample. The dendritic structure of the weld line between the WZ and FZ is more uniform compared to that of the A500-T91 sample. The heat input required for welding with austenitic filler wires is significantly lower than that for ferritic filler wires. Figure 6b shows the formation of martensite phases in the CGHAZ due to the cooling rate in the WZ. Figure 6c depicts the microstructure of the FGHAZ, revealing fine and homogeneous grain structures that enhance the overall strength of the material. The bright small dots visible in the microstructure represent carbides, which significantly contribute to the hardness and high temperature strength of the material. Figure 6d shows the ferrite, martensite-austenite (M/A) components, and martensite formation in the ICHAZ, where grain growth occurs. In zones dominated by these structures, the material exhibits ductile and soft phases, leading to the lowest hardness in this zone. Figure 6e demonstrates the microstructural transformation along the SCHAZ and BM, characterized by a gradual phase transition. The reformed martensitic structures in this zone contribute to the gradual increase in material hardness. Additionally, if thin linear features are observed in the SEM images, they represent carbide precipitates formed after welding. These carbides are found within the ferritic phase or surrounding the M/A components.

### 3.2. Hardness Results

**Figure 7:**
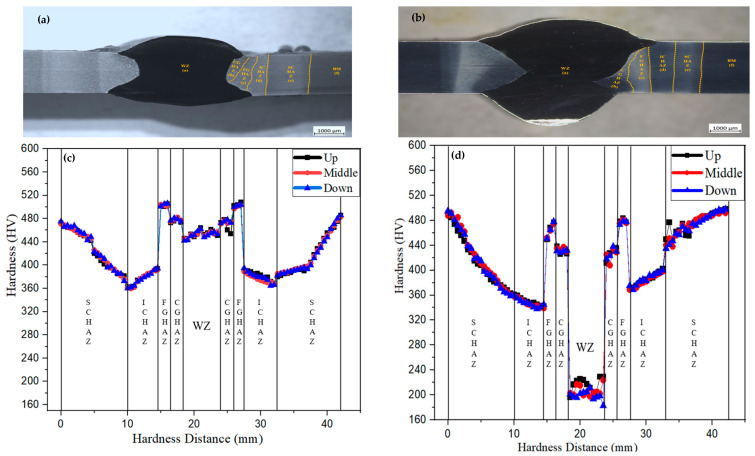
Hardness test samples and results graphics of (**a,c**) A500-T91 and (**b,d**) A500-ER371.

The effects of austenitic and ferritic filler wires on the hardness distribution of A500 bulletproof steel plates were evaluated across three critical zones: the BM, HAZ, and WZ, as presented in Figure 7. The WZ of the T91 filler demonstrated higher hardness, attributed to the formation of a martensitic matrix, which facilitated strength matching with the BM. Conversely, the WZ of the ER371 filler exhibited lower hardness due to the presence of an austenitic matrix, which inherently contributes to reduced hardness.

In the WZ, the T91 filler reached an approximately hardness of 470 HV1, while the ER371 filler reached an approximately hardness of 185 HV1. In the CGHAZ, the T91 filler achieved a hardness of 480–485 HV1, while the ER371 filler produced a lower hardness of 435–440 HV1. The highest hardness value for T91, approximately 518 HV1, was recorded in the FGHAZ. This significant hardness increase was attributed to the formation of a martensitic matrix, which is inherently hard, combined with a fine-grained structure that enhanced the material’s overall strength. On the other hand, the FGHAZ hardness for ER371 was slightly lower than that of T91, mainly due to the presence of an austenitic structure, which is less hard than martensite. In this zone, the highest hardness was measured at 480 HV1.

In the ICHAZ, the hardness distribution displayed a distinct trend for both fillers. For the T91 filler, the hardness decreased progressively from approximately 400 HV1 near the FGHAZ to 360 HV1 toward the SCHAZ. This softening is indicative of the formation of a two phase zone, with ferrite and RA as the dominant phase, which is inherently softer. Additionally, the SCHAZ experienced a tempering like effect due to high temperature exposure, leading to a reduction in the hardness of the martensitic matrix.

For the ER371 filler, the hardness in the ICHAZ showed a significant decrease at the point where it intersected with the FGHAZ, followed by a gradual increase towards the SCHAZ. For the ER371 weld, the lowest hardness was recorded as 330 HV1 in the ICHAZ, while for T91, the lowest hardness was recorded as 360 HV1 at the intersection of the ICHAZ and SCHAZ. For both fillers, an increasing trend in hardness was observed moving from the SCHAZ to the BM, highlighting the gradient nature of the microstructural transitions in these zones [16].

The CGHAZ forms adjacent to the weld metal due to exposure to high peak temperatures and is characterized by coarse-grained primary austenite and coarse-grained ferrite. In the ICHAZ, phase transformations include the partial decomposition of martensite into ferrite and bainite, accompanied by the formation of M/A constituents due to localized thermal gradients. The SCHAZ, on the other hand, is characterized by the formation of over-tempered martensite as a result of prolonged exposure to elevated temperatures, before transitioning into the BM [17].

**Figure 8:**
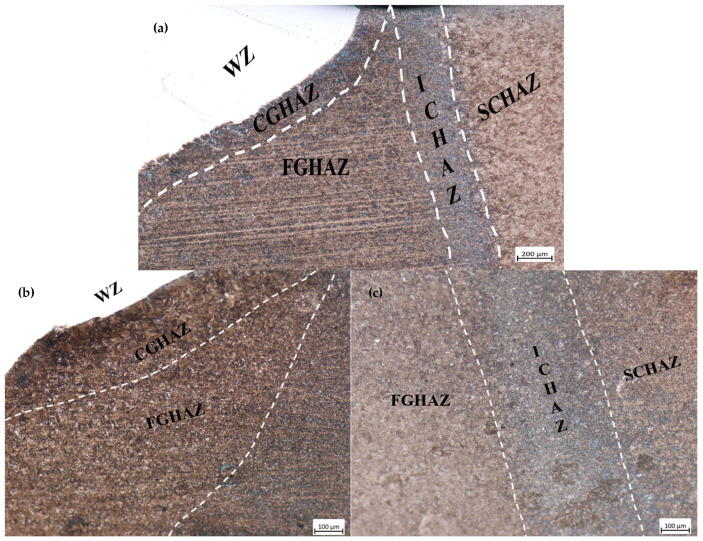
OM microstructure of A500 welded with different welding consumables: (**a**) ER371; (**b**,**c**) T91.

Figure 8 shows the formation and microstructural characteristics of the FGHAZ and ICHAZ for both types of welding consumables, highlighting their distinct phase compositions. The formation of the ICHAZ primarily results in a microstructure dominated by ferrite, which is softer than other phases and significantly influences the hardness in this zone. While M/A constituents may appear in limited amounts, their contribution remains secondary compared to the dominant ferritic structure. The dimensions and dispersion of these M/A particles differ between the ER371 austenitic welding filler metal and the T91 ferritic filler metal, leading to variations in local hardness levels. For austenitic welding filler wires like ER371, the unique microstructure and reduced thermal conductivity contribute to localized thermal accumulation, which can result in distinct hardness profiles. However, the microstructural transformations primarily favor softer phases, such as ferrite, rather than hardness enhancing phases like martensite. Conversely, the T91 filler metal predominantly yields fine-grained tempered martensite in the FGHAZ, which directly enhances hardness in this zone [12].

### 3.3. Tensile Results

The tensile properties of A500 ballistic steel joints, welded with ER371 and T91 welding consumables, were thoroughly investigated. It was observed that the yield point was not distinctly defined in any of the tested samples. As delineated in Table 5, both the tensile strength and the percentage of elongation for the welded specimens were found to be inferior to those of the base material.

**Figure 9:**
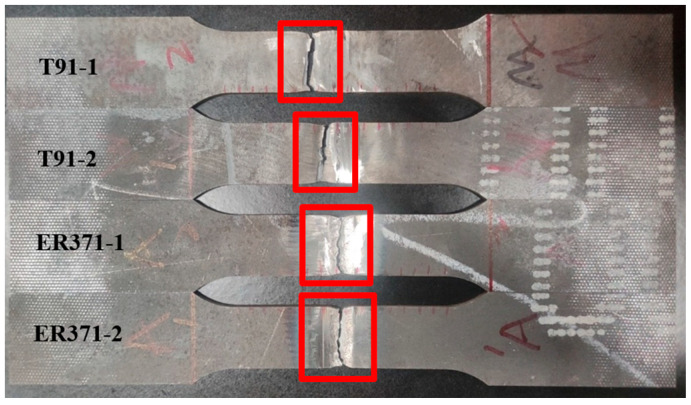
Tensile fracture position for T91 and ER371.

Figure 9 shows the tensile fracture surfaces of A500 ballistic steel joints welded with T91 and ER371 filler wires, with the fracture zones of each specimen (T91-1, T91-2, ER371-1, and ER371-2) highlighted in red boxes.

The fracture surfaces of the T91 welded specimens exhibited characteristics of brittle fracture, characterized by sharp and relatively flat fracture planes. This behavior is attributed to the predominant martensitic microstructure formed during welding with the T91 ferritic filler wire. These observations align with the previously discussed high hardness values recorded in the FGHAZ, further emphasizing the brittle nature of the fracture.

In contrast, the ER371 welded specimens demonstrated a distinctly different fracture behavior. The fractures were localized within the WZ, indicative of the austenitic microstructure developed during welding with the ER371 filler wire. This microstructure provides superior toughness and ductility compared to the T91 joints, resulting in improved resistance to brittle failure [18].

**Figure 10:**
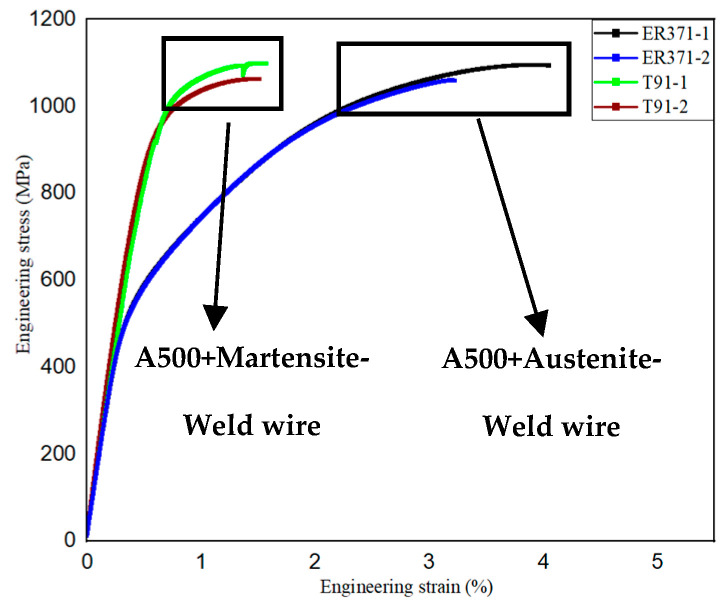
Engineering stress–strain curves.

Figure 10 shows the engineering stress–strain curves, which further reveal that the T91 filler material exhibits a higher yield strength of 989 MPa, but comparatively lower elongation due to its brittle fracture characteristics.

Conversely, the ER371 filler material demonstrates greater ductility, as evidenced by its higher elongation and superior toughness. Although its tensile strength is comparable to that of the T91 filler material, the yield strength of ER371 is significantly lower, at 624 MPa. In the analysis conducted by Ö.S. Bölükbaşı et al., the tensile test results of Armox 500T steel welded using Esab Ok Autrod 16.95 wire with the GMAW process were comparable to those of A500 steel welded with ER371 filler. Although the A500-ER371 joint exhibited slightly higher elongation, its tensile strength was lower. However, the overall values remained comparable [19].

The reason for the difference in the fracture location is that the microstructure differences in the two WZs and the heat effect zone led to performance differences. The WZ of T91 has a martensitic structure, the strength of which is similar to the base material, but due to the higher welding heat input, the grains in the heat effect zone grew, which causes a serious decrease in performance. On the other hand, the WZ of ER371 has an austenitic structure, and the strength of this zone is the lowest among all zones; therefore, the fracture occurs in the WZ [20,21].

### 3.4. Impact Results

**Figure 11:**
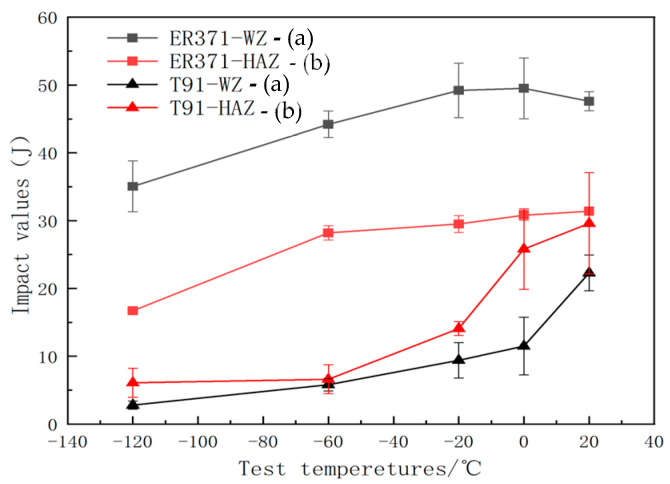
WZ (a) and HAZ (b) results of ER371/T91 impact energy test.

Figure 11 shows the graphs depicting the impact test results of welded specimens joined with A500 steel. The graphs reveal that the impact energy is greater for the WZ and HAZ in ER371 compared to T91. This increased impact energy is attributed to the higher level of toughness and ductility exhibited by the A500 steel when joined with austenitic wire.

**Figure 12:**
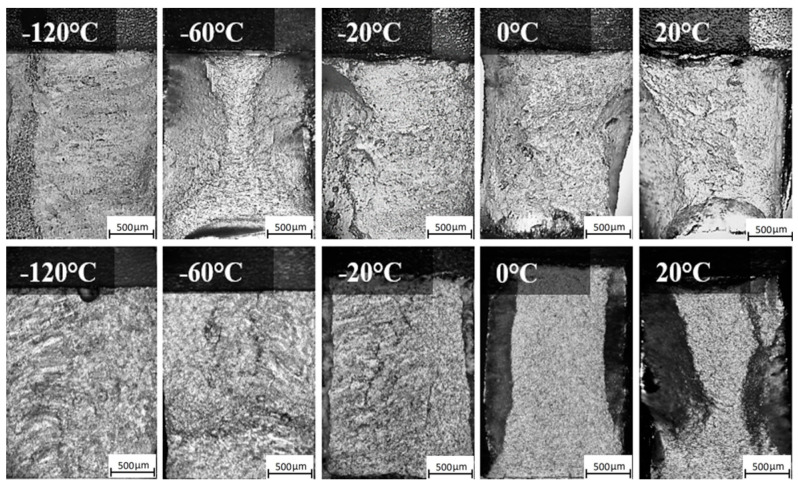
Macroscopic image of the fracture surfaces of representative samples of WZ A500-ER371 (**top**) and A500-T91 (**bottom**).

**Figure 13:**
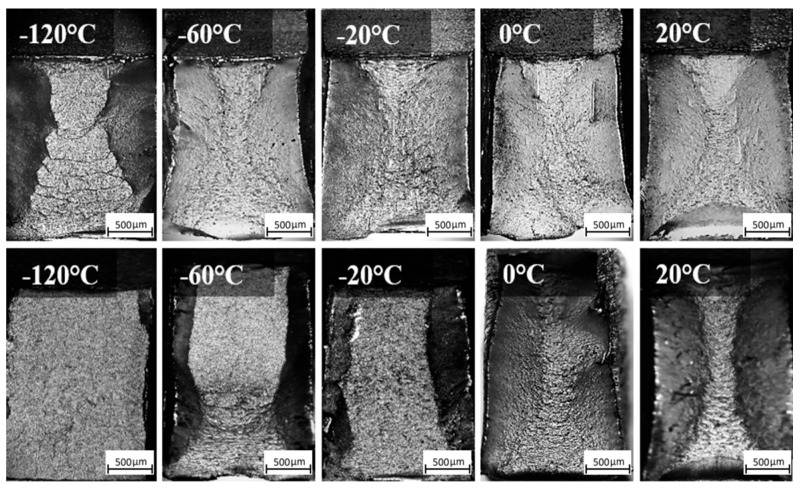
Macroscopic image of the fracture surfaces of representative samples of HAZ A500-ER371 (**top**) and A500-T91 (**bottom**).

Figure 12 and Figure 13 show macroscopic images of the fracture surfaces of ER371 and T91 welds following the impact toughness test. As seen in Figure 12, at 20 °C, the WZ surface of the base metal welded with ER371 austenitic filler metal reveals typical high toughness fracture features, such as a significant fibrous appearance and rounded features that are characteristic of ductile fracture zones. These observations indicate that the filler material’s austenitic structure contributed to the fracture morphology, achieving the highest measured impact energy of 55 J. As the temperature decreased, the fracture surface exhibited a reduction in fibrous morphology, and at −120 °C, the fracture became more brittle, with the lowest recorded impact energy of 35 J.

For the T91 welding consumable, shear lip zones were observed, with the shear lip area decreasing as the temperature was lowered. The fracture surface of the T91-WZ specimens exhibited less roughness overall, correlating with lower impact energy compared to ER371-WZ. At lower temperatures, the fracture surfaces became progressively smoother, with shear lip zones disappearing entirely, indicating a transition to brittle fracture behavior [22,23].

Figure 13 shows macroscopic images of the fracture surfaces in the HAZ subjected to the impact test. A clear distinction is observed between the fracture surfaces of welds filled with austenitic (ER371) and ferritic (T91) materials. In ER371-HAZ specimens, the shear lip zones are less pronounced, accompanied by a comparatively rougher fracture surface. Conversely, T91-HAZ specimens display more defined shear lip zones and smoother fracture surfaces. This variation in fracture morphology indicates that the ER371 filler provides higher toughness, as evidenced by the distinct residual deformation patterns visible on ER371-HAZ fracture surfaces, which correlate with greater resistance to impact energy absorption. Furthermore, ER371-WZ exhibits superior impact resistance compared to ER371-HAZ, with noticeable differences in fracture roughness due to variations in microstructural deformation mechanisms. At 20 °C, the impact energy for ER371-HAZ reaches a maximum of 32.1 J. However, as the temperature decreases, both the visible deformation patterns and impact energy diminish, with the lowest value recorded at 16.4 J at −120 °C [24].

In contrast, T91-HAZ specimens demonstrate temperature-sensitive fracture behavior, with shear lip zones becoming progressively less distinct as temperature decreases, a trend similar to that seen in T91-WZ. The transition to brittle fracture for T91-HAZ occurs at −20 °C, whereas ER371-HAZ retains ductility and toughness down to −120 °C. This significant difference in the brittle transition temperature highlights the superior impact energy retention capability of ER371-HAZ compared to T91-HAZ.

### 3.5. Ballistic Performance

This study investigates the ballistic response of A500 steel joints welded with austenitic (ER371) and ferritic (T91) filler materials under the impact of 5.56 mm × 45 mm NATO Ball (SS109) bullets. Harder materials typically exhibit superior resistance to bullet penetration. In accordance with STANAG 4569, a NATO standard defining protection levels for occupants of logistical and lightly armored vehicles, Level 1 protection mandates resistance against SS109 bullets traveling at 900 ± 20 m/s from a distance of 30 m. The standard stipulates that no visible cracks should form on the target plate and that no spalling or material fragmentation should occur on the rear side. Complete Penetration (CP) is characterized by the bullet, its fragments, or target debris perforating the witness plate, as confirmed by visible light penetration, whereas Partial Penetration (PP) occurs when the impact does not result in light visibility through the witness plate.

**Figure 14:**
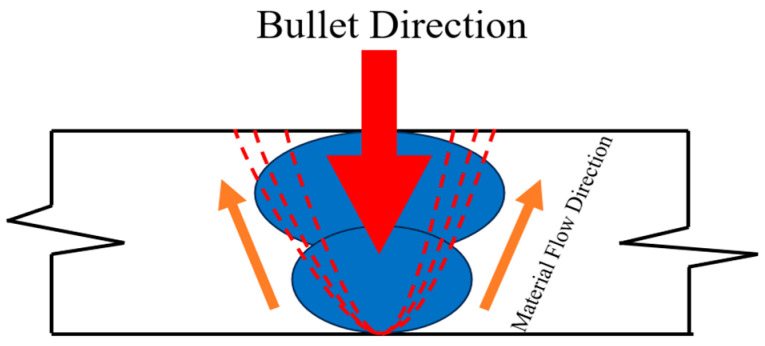
Schematic drawing shows the possible solid-state flow of the material during impact. (Arrows indicate the flow direction of the material, and the dotted line represents the ASS buttering layer).

A schematic representation of the bullet impact mechanism is provided in Figure 14, illustrating the material flow and energy dissipation upon bullet impact. The red arrow denotes the bullet’s trajectory, while the blue regions indicate zones of concentrated stress and deformation. The orange arrows depict the material flow direction resulting from the impact. The observed outward material flow, perpendicular to the target surface, is attributed to the presence of a high hardness BM adjacent to the softer SS buttering layer. [25]. This difference in hardness likely contributes to the restriction of material displacement in the direction perpendicular to bullet penetration and parallel to the target surface, thereby influencing the overall ballistic performance of the welded joint.

**Figure 15:**
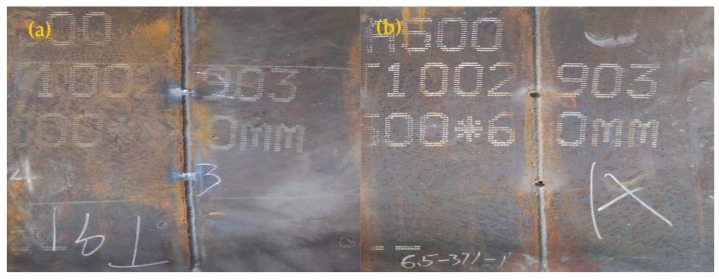
Penetration test plates of (**a**) A500-T91 and (**b**) A500-ER371.

A comparative analysis of Figure 15a,b underscores the critical influence of filler material selection on the ballistic performance of welded joints. The martensitic T91 welding wire exhibits significantly greater resistance to bullet penetration, effectively mitigating the impact and preventing complete failure. In contrast, the austenitic ER371 welding wire demonstrates insufficient ballistic resistance, failing to prevent full penetration. This discrepancy highlights the superior performance of ferritic welds in ballistic applications.

**Figure 16:**
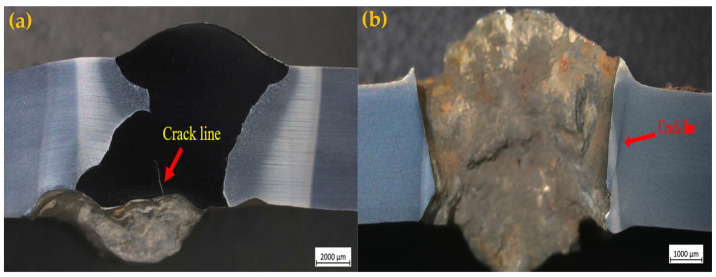
After SS109 bullet penetration crack. (**a**) A500-T91; (**b**) A500-ER371.

Macrostructural analysis of bullet impact damage is presented in Figure 16, illustrating the failure characteristics of A500-ER371 and A500-T91 welded samples. The T91 sample exhibits the formation of microcracks near the penetration site, with minor crack propagation towards the weld zone (WZ). However, the bullet is effectively halted, and no catastrophic failure is observed. Conversely, the ER371 sample experiences complete penetration, exhibiting substantial material deformation and an extensive damage zone surrounding the impact site. These findings indicate a pronounced difference in ballistic behavior, with the T91 weld providing superior penetration resistance.

For standardized damage assessment, a grading system was implemented, where damage levels 1–4 represented acceptable conditions, while levels 5–8 denoted unacceptable failure. The austenitic ER371 welds were classified as Level 8, indicating complete penetration and severe damage, whereas the ferritic T91 welds were rated as Level 2, with only minor back face bulging. These results confirm that ferritic filler materials offer superior ballistic resistance compared to their austenitic counterparts, reinforcing the importance of filler material selection in armor steel applications.

## 4. Conclusions

In this study, the microstructure and mechanical properties of the welded and weld-affected zones of A500 bulletproof steel, welded using austenitic ER371 and ferritic T91 welding wires with the GMAW method, were analyzed. The aim is to contribute to the advancement of welding techniques and filler materials used in armor steels, supporting efforts to enhance both the structural integrity and ballistic resistance of welded joints.

The authors acknowledge the dual-use potential of the findings and confirm that all necessary precautions have been taken to prevent misuse. Adherence to national and international regulations on Dual Use Research of Concern (DURC) has been maintained, ensuring ethical responsibility in the deployment of this technology.

1. In the A500-ER371 weld, the WZ exhibited the lowest hardness of 185 HV1 due to its austenitic structure, while the CGHAZ displayed a hardness of 440 HV1, attributed to martensite phases formed during cooling. The FGHAZ reached a peak hardness of 480 HV1, comprising martensite, bainite, and ferrite phases, with carbides enhancing strength. The ICHAZ showed a hardness drop to 330–360 HV1, dominated by ferrite and M/A phases. Conversely, in the A500-T91 weld, the WZ achieved higher hardness at 470 HV1, due to a martensitic matrix. The CGHAZ had a hardness of 485 HV1, reflecting the presence of martensite and fine-grained ferrite, while the FGHAZ exhibited the highest hardness of 515 HV1, attributed to its dominant martensitic structure and fine-grained microstructure. The ICHAZ experienced softening with hardness reducing to 360 HV1, due to ferritic structures and tempering effects. In both welds, a gradual increase in hardness was observed when transitioning from the SCHAZ to the BM.
2. A500-T91 welds demonstrated higher tensile and yield strengths (1099 MPa and 1026 MPa, respectively), but lower elongation (2%), indicating brittle fracture behavior. Conversely, A500-ER371 welds exhibited lower tensile strength (1077 MPa) and yield strength (624 MPa), but higher elongation (5%), reflecting a more ductile fracture mechanism. The impact test results highlighted the superior toughness and impact energy absorption of ER371, which maintained ductility down to −120 °C, while T91 transitioned to brittle fracture at −20 °C.
3. The ballistic tests revealed that welds with T91 ferritic wire showed superior resistance to bullet penetration, attributed to the martensitic structure. In contrast, welds with ER371 austenitic wire exhibited weaker performance, allowing bullet penetration. The microstructural characteristics of the two welding wires played a key role, with T91’s martensitic matrix contributing to higher hardness and strength, which improved the material’s ability to resist deformation under impact. The ER371 welds, with their softer austenitic structure, failed to resist bullet penetration due to their lower hardness.

## Figures and Tables

**Table 1:** Chemical composition of base metal and filler metals (wt%).

Materials		C	Si	Mn	P	S	Cr	Ni	V	Mo	Nb	Other Ingredients
Base Metal	A500	0.25	0.16	0.79	0.015	0.004	0.90	0.92	0.07	0.21	0.053	≤2.00
Welding Consumables	T91	0.10	0.25	0.61	0.006	0.001	9.0	0.55	0.21	0.98	0.079	≤2.00
	ER371	0.09	0.81	7.13	0.019	0.008	19.02	8.35	0.2	0.025	-	≤2.00

**Table 2:** Welding procedure parameters.

Weld Consumables	Protective Gas	Gas Flow L/min	Interlayer (°C)	Welding Current (A)	Welding Voltage (V)	Welding Speed (mm/s)
MIG-ER371	Ar/CO_2_	20	88	122	18.6	5.4
T91	Ar/CO_2_	20	84	140	18.2	4.2

**Table 3:** A500 steel (Pennsylvania/USA/Vickers/HV1) hardness measurement.

A500/mm	1	2	3	4	5	6	7	8	9	10	Average
4.1	498	501	511	513	518	512	508	509	499	516	509

**Table 4:** Armox 500T mechanical properties of armor steel.

Hardness	Yield Strength (Mpa)	Tensile Strength (Mpa)	Elongation (%)	Impact Energy (−40/J)
480–540 HB	1250 Mpa	1450–1750 Mpa	10%	25 J

**Table 5:** Tensile mechanical properties.

		Tensile Strength (MPa)/CV (%)	Yield Strength (MPa)/CV (%)	Elongation (%)/CV (%)
Base	A500	1700/5.0	1560/5.0	10.3/4.8
Welding consumables	ER371	1095/5.0	624/4.9	4.9/4.1
	T91	1062/4.9	989/4.9	2.1/4.8

## Data Availability

The original contributions presented in the study are included in the article; further inquiries can be directed to the corresponding author.
